# A review of the literature on the accuracy, strengths, and limitations of visual, thoracic impedance, and electrocardiographic methods used to measure respiratory rate in hospitalized patients

**DOI:** 10.1111/anec.12885

**Published:** 2021-08-18

**Authors:** Linda K. Bawua, Christine Miaskowski, Xiao Hu, George W. Rodway, Michele M. Pelter

**Affiliations:** ^1^ School of Nursing University of California San Francisco California USA; ^2^ School of Nursing Duke University Durham North Carolina USA; ^3^ School of Medicine University of Nevada Reno Nevada USA

**Keywords:** electrocardiography, hospitalized patients, impedance pneumography, respiratory rate, sensitivity/specificity, visual assessment

## Abstract

**Background:**

Respiratory rate (RR) is one of the most important indicators of a patient's health. In critically ill patients, unrecognized changes in RR are associated with poorer outcomes. Visual assessment (VA), impedance pneumography (IP), and electrocardiographic‐derived respiration (EDR) are the three most commonly used methods to assess RR. While VA and IP are widely used in hospitals, the EDR method has not been validated for use in hospitalized patients. Additionally, little is known about their accuracy compared with one another. The purpose of this systematic review was to compare the accuracy, strengths, and limitations of VA of RR to two methods that use physiologic data, namely IP and EDR.

**Methods:**

A systematic review of the literature was undertaken using prespecified inclusion and exclusion criteria. Each of the studies was evaluated using standardized criteria.

**Results:**

Full manuscripts for 23 studies were reviewed, and four studies were included in this review. Three studies compared VA to IP and one study compared VA to EDR. In terms of accuracy, when Bland–Altman analyses were performed, the upper and lower levels of agreement were extremely poor for both the VA and IP and VA and EDR comparisons.

**Conclusion:**

Given the paucity of research and the fact that no studies have compared all three methods, no definitive conclusions can be drawn about the accuracy of these three methods. The clinical importance of accurate assessment of RR warrants new research with rigorous designs to determine the accuracy, and clinically meaningful levels of agreement of these methods.

## 1 BACKGROUND

Assessment of respiratory rate (RR) is often neglected when vital signs are obtained in hospitalized patients, which is problematic given that unrecognized changes in RR are associated with worse patient outcomes (Brekke et al., 2019; Kelly, 2018; Mochizuki et al., 2017; Subbe & Kinsella, 2018) including increases in cardiopulmonary arrest and in‐hospital mortality (Cretikos et al., 2007, 2008; Fieselmann et al., 1993; Goldhill et al., 2005; Subbe et al., 2003). An abnormal RR is observed in a wide range of both acute and chronic conditions (Philip et al., 2015). Therefore, early detection of changes in RR and abnormal breathing characteristics (e.g., depth, use of accessory muscles, skin color) can be used to determine a patient's health status, aid in the selection of appropriate treatments, and determine when a patient is ready to transition from a high to a sub‐acute level of care or discharge from the hospital. The assessment and documentation of vital signs in hospitalized patients have been noted to be deficient (Cretikos et al., 2008; Leuvan & Mitchell, 2008). Of the four vital signs (i.e., RR, heart rate, blood pressure, temperature), RR is the one that is most frequently missing in the medical record, even when the patient's primary diagnosis is respiratory‐specific (Cretikos et al., 2008). Reasons cited include the length of time required to obtain this measure and the interruptions created in workflow efficiency (Kelly, 2018; Nielsen et al., 2015). In some unstable patients, dynamic fluctuations in RR are even more significant than changes in systolic blood pressure or heart rate, which suggests that RR may be a better indicator of physiologic instability (Cretikos et al., 2008; Leuvan & Mitchell, 2008).

### 1.1 Assessment of RR in hospitalized patients

In hospitalized patients, abnormal RR (e.g., tachypnea, bradypnea) are indicators of respiratory instability, respiratory compromise, and often the first indication of impending respiratory arrest and/or the need for rescue intubation (Cretikos et al., 2007, 2008; Goldhill et al., 2005). However, identifying these acute changes can be delayed and/or missed if RR is not obtained often and with a high degree of accuracy. Therefore, assessing RR at more frequent intervals and more accurately may lead to earlier detection of clinical deterioration and appropriate intervention(s) to improve patient outcomes. To achieve this goal, the ideal method to assess RR would be accurate, sensitive, specific, non‐invasive, and affordable; use currently available physiologic data; and easily be integrated into clinical care environments with minimal disruption. While end‐tidal CO2 is the gold standard device‐driven method, this method is used primarily in the operating room, cardiac catheterization laboratory, and in some emergency departments. However, this technique has not been applied broadly in the intensive care unit, which is the focus of this review. Current World Health Organization recommendations state that measurement of RR should include a 60‐s visual count, or auscultation for the number of breaths taken, because it is the most reliable method and noted that no other gold standard measure exists (WHO, 1990). While visual assessment (VA) of RR is recommended, several hospital‐based studies found that RR is often not assessed, and even when recorded in the health record, it is often inaccurate (Cretikos et al., 2008; Kamio et al., 2018; Kelly, 2018). Surprisingly, even among patients whose primary diagnosis is respiratory, assessment of RR is often not accurate (Badawy et al., 2017; Cretikos et al., 2008; Hogan, 2006; McGaughey et al., 2007).

Several challenges specific to the hospital setting make accurate RR assessment challenging. For example, nurses report that the VA of RR is one of the most challenging nursing tasks (Kelly, 2018; Nielsen et al., 2015). Another study found that clinicians believe that this time‐consuming procedure does not provide useful clinical information, especially when RR is challenging to obtain (e.g., agitated or uncooperative patients) (Kamio et al., 2018). In addition, the VA of RR can be interrupted by conversations or other distractions. These obstacles and clinicians' opinions about the clinical utility of carefully measuring RR have contributed to the above‐outlined problems and highlight how continuous and non‐invasive methods may improve RR assessment.

### 1.2 Purpose statement

The purpose of this literature review was to compare the accuracy, strengths, and limitations of VA of RR to two methods that use physiologic data, namely impedance pneumography (IP) and electrocardiographic‐derived respiration (EDR). The next sections of this article describe each of these methods.

### 1.3 Visual assessment (VA)

Visual assessment of RR is performed by asking a patient to lie still and refrain from talking. Then, the clinician counts the number of times the chest rises and falls for a full minute (Wheatley, 2018). In addition to counting the number of respirations, this method involves assessing the patient's skin and mucous membranes for color, moisture, temperature, and breathing characteristics (e.g., depth, nasal flaring, use of accessory muscles). This method requires concentration and can be difficult if a patient cannot follow instructions and/or cooperate. While RR is a critical determinant of a patient's current physiologic state (Cretikos et al., 2007, 2008; Fieselmann et al., 1993) VA of RR is often estimated, guessed, or omitted altogether (Cooper et al., 2014). In one study (Ansell et al., 2014), the nurses surveyed reported intentionally or unintentionally omitting RR assessment >90% of the time. In another study (Leuvan & Mitchell, 2008), of 62 patients with 1597 unique vital signs recorded, only one reading per day of RR was recorded compared with 5.0 for blood pressure; 4.4 for heart rate; and 4.2 for temperature (all *p *< .001). Incorrect RR readings (low or high) can occur during routine patient activities such as talking, turning, or moving in bed (Krapohl & Shaw, 2015). Finally, in some cases, clinicians reported that they simply copy a previous RR rather than do a VA (Cooper et al., 2014).

### 1.4 Impedance pneumography (IP)

Evaluation of electrical impedance in body tissues is a common technique that uses variability in tissue volumes to measure the resistance of alternating currents (AC) as electricity travels through a given material (Yanovski et al., 1996). Measurement of impedance is used in several body composition assessments (e.g., body fat, muscle mass) (Yanovski et al., 1996). In the hospital setting, the IP method uses the same skin electrodes to measure both the ECG and RR. It should be noted that while ECG lead wires and skin electrodes are used for the IP evaluation of RR, ECG waveforms are not used to calculate RR. Rather, the ECG device (through lead wires attached to skin electrodes) directs a very small amount of electrical current into the patient's body, that is measured as electrical impedance (Ansari et al., 2016; Gupta, 2011).

**FIGURE 1:**
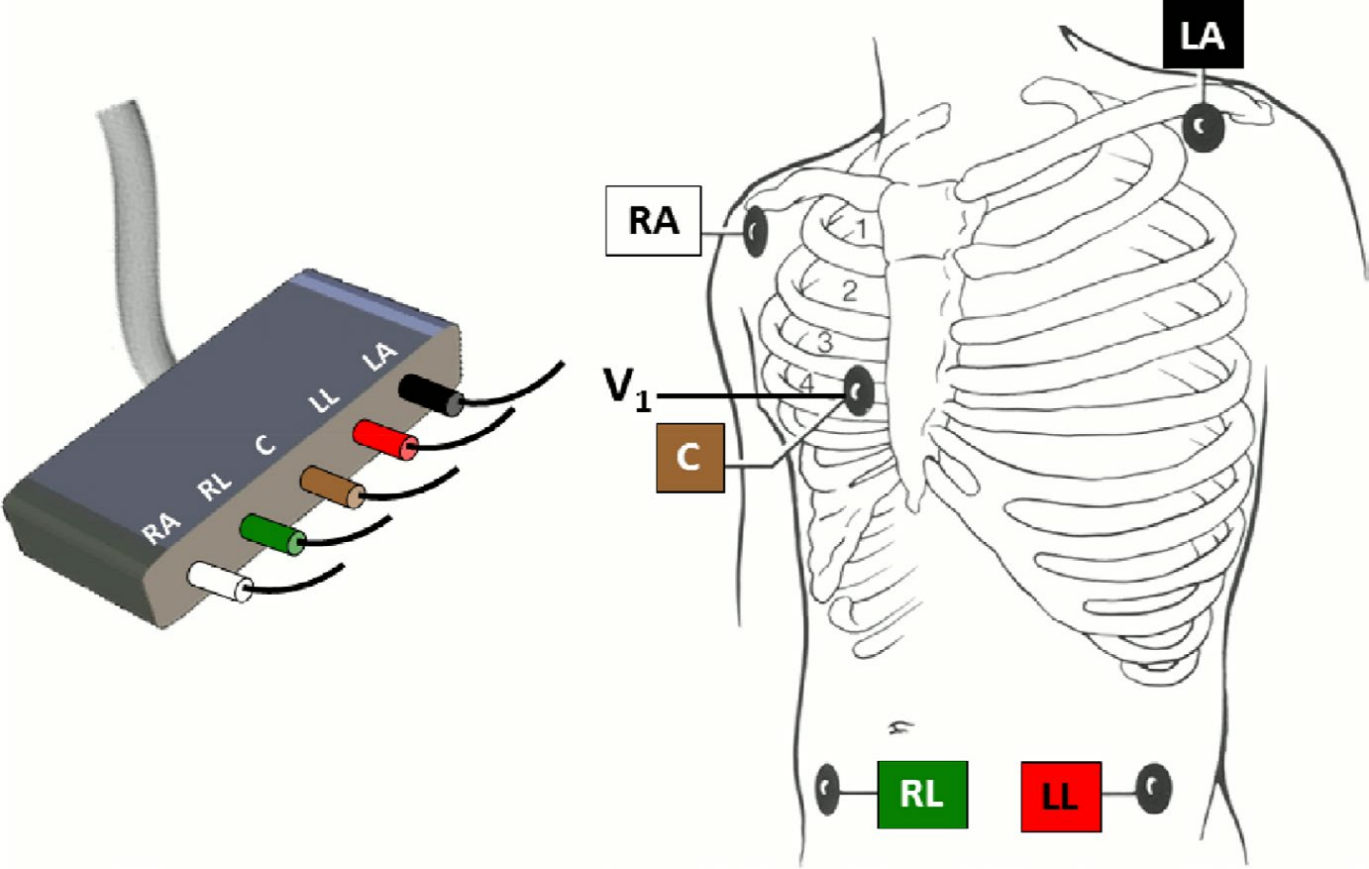
An Illustration of how electrocardiographic (ECG) limb leads I, II, and III are obtained using skin electrodes placed on the right arm (RA), left arm (LA) and left leg (LL). Impedance respiration is typically generated using one or two of these ECG leads using the bedside monitor. A single chest (C) electrode is shown that is routinely placed in the V_1_ position for in‐hospital arrhythmia monitoring and the right leg (RL) electrode, that is required to record lead V_1_. Lead V_1_ is not used for deriving respirations. Figure from Drew et al., PLoS One https://doi.org/10.1371/journal.pone.0110274.g003 (Drew et al., 2014)

Depending on the manufacturer, one or two of the limbs leads or a combination of two are used to detect amplitude differences of the injected current (Figure 1). During inspiration, as the chest expands, resistance to the flow of an electrical current increases, which increases impedance. Alternatively, during expiration, impedance decreases as air leaves the lungs. To derive RR using the IP method, a drive‐and‐measure circuit is established that delivers two out‐of‐phase AC‐coupled currents onto a combination of electrodes (Gupta, 2011; Redmond, 2013).

**FIGURE 2:**
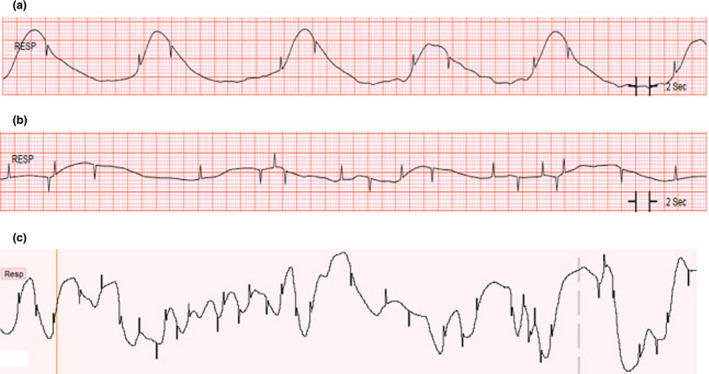
(a–c) Accurate (a), inaccurate (b), and motion artifact (c) respiratory waveforms using the impedance pneumography (IP) respiration method. (a) Normal respirations are generated from a 10 s IP waveform. Note the upward flag on the inspiratory waveform and the downward flag on the expiratory waveform, which are added by this particular manufacturer (GE Healthcare). (b) Inaccurate respiratory rate from a 10 s IP waveform recording. Note that occurrence of indistinguishable waveforms that are indicative of inspiration and expiration and the random flags throughout the tracing. (c) An illustration of a 20 s IP waveform during motion artifact, which resulted in an alarm for a respiratory rate of 55 breaths/min. Note that flags are present on the tracing that coincide with the oscillations of the IP waveform

A series of resistors and capacitors send a very low amplitude current into the patient's chest via the ECG lead wires (Gupta, 2011; Redmond, 2013). Given that the AC is minimal, patients do not experience any adverse effects, or experience any sensations associated with the injected current. A computer algorithm within the bedside ECG monitor generates both a numeric RR (breaths/minute) and a respiratory waveform. An accurate IP waveform is shown in Figure 2a.

Several caveats warrant consideration regarding the IP method. For example, the best lead(s) to obtain an accurate RR in a person who is an abdominal breather are typically lead II and/or lead III (Redmond, 2013). These two ECG leads make sense for this application because lead II is obtained using the right arm and left leg electrodes and lead III is obtained using the left arm and left leg electrodes; thus, thoracic changes associated with abdominal breathing are most noticeable using these two leads. However, if a patient is in an upright position, or a chest breather, a more accurate ECG lead for RR detection may be lead I, which uses the right arm and left arm electrodes. For this reason, the ideal IP algorithm for hospitalized patients should use a combination of multiple ECG leads to derive the most accurate RR. However, few IP algorithms use multiple ECG leads or have the ability to adjust automatically to changes in body position (Varon et al., 2020). Lastly, regardless of which ECG lead is used for RR detection, any one of these leads can be contaminated by poor skin electrode contact, inadvertent ECG lead swap (i.e., limb leads used for IP signal) motion artifact caused by pulling or pressing on the skin electrodes used to generate the IP signal, or disconnected lead(s), making the IP method prone to inaccurate RR measurement (Ansari et al., 2016). Figure 2b,c are examples of contaminated IP signals.

### 1.5 Electrocardiographic‐derived respiration (EDR)

The graphic display of the heart's electrical activity provided by the ECG can be used to estimate RR. The EDR method uses the ECG waveforms recorded from the lead wires placed on a patient's chest to detect subtle variability in QRS morphology and timing during breathing (Moody et al., 1985).

The IP method detects subtle variability in QRS morphology and timing that are generated by changes in both lung volume and the heart's position relative to the ECG leads on the body's surface (AL‐Khalidi et al., 2011; Helfenbein et al., 2014; Larsen et al., 1984). Unlike IP, the EDR method uses direct assessment of respiratory‐influenced variations in morphology and timing over a series of consecutive QRS complexes to derive RR. The EDR algorithms typically use direct assessment of respiratory‐influenced variations in morphology and timing over a series of consecutive QRS complexes (Helfenbein et al., 2014; Lazaro et al., 2014; Moody et al., 1985; Orphanidou et al., 2013). Several different algorithms are used to estimate RR from single and/or multi‐lead ECG waveform morphologies (Behbehani et al., 2002; De Chazal et al., 2003; de Geus et al., 1995). Two of these algorithms (i.e., respiratory sinus arrhythmia [RSA], respiratory amplitude modulation [RAM]) are discussed in more detail below (Helfenbein et al., 2014).

#### 1.5.1 EDR method using respiratory sinus arrhythmia (RSA)

**FIGURE 3:**
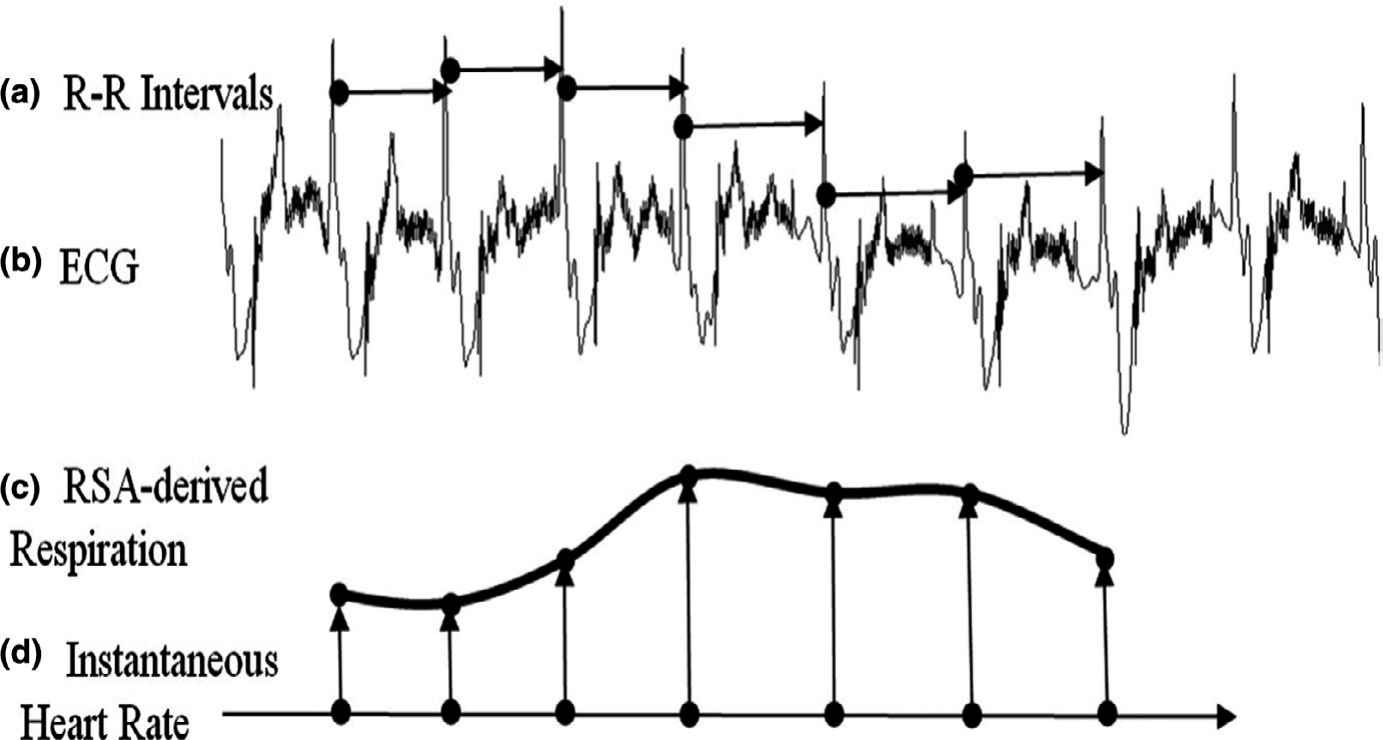
An illustration of a respiratory sinus arrhythmia (RSA) derived respiratory rate (a,b), which uses varying RR intervals (horizontal arrows) from QRS complexes on the electrocardiogram (ECG). Note that the circles and arrowheads of the horizontal arrows de‐note the QRS complexes. The inverse of the RR intervals is shown as vertical arrows (d), which are exaggerated for illustration. A heart rate is computed, which is used as amplitude knots for cubic spline interpolation to create the RSA‐derived respiration waveform (c). Reprinted with permission from the journal (Helfenbein et al., 2014)

During inspiration and expiration, the heart rate slightly increases and then decreases. This phenomenon is referred to as RSA and is depicted in Figure 3 (Helfenbein et al., 2014). The amount of respiratory oscillation differs from person to person and varies depending on the rate of an individual's breathing (e.g., tachypnea, bradypnea) (Charlton et al., 2016).

Because of the response of the autonomic nervous system to the baroreflex sensors in peripheral arteries, which respond to minor changes in blood pressure induced by oscillations of thoracic pressure from the respiratory cycle, instantaneous changes in heart rate, a computation of heart rate variability and its inverse (RR interval) can be used to derive the rhythm of an individual's respiration (Helfenbein et al., 2014).

#### 1.5.2 EDR method using RAM

This algorithm takes advantage of anatomic movements related to respiration that affect the ECG. First, the heart's apex extends toward the abdomen as it stretches during inspiration and simultaneously the diaphragm moves downward (Lazaro et al., 2014). Second, during exhalation the diaphragm recoils to aid in emptying the lungs and squeezes the heart's apex toward the sternum. During these processes, compared with a reference vector, the angles of the electrical and cardiac vectors are altered. These alterations exert a modifying influence on the amplitude of the ECG signals that are used to identify respirations (Moody et al., 1985). Recently, the RAM algorithm was simplified using total (peak‐to‐trough) QRS amplitude in a single lead (Helfenbein et al., 2014). This modified process includes the following steps: (1) detection of QRS complexes; (2) measurement of the total QRS amplitude; (3) exclusion of outliers (e.g., noise and artifacts); (4) interpolation of the EDR values, and (5) separation of the waveform with a band‐pass filter as suited for the range of rates anticipated (Helfenbein et al., 2014).

## 2 METHODS

For this review, a systematic literature search was conducted using the following databases: PubMed, Cumulative Index to Nursing and Allied Health Literature (CINAHL), Web of Science, and the Cochrane Library. Keywords used for the database searches included: *adult(s)*, *respiration(s)*, *RR measurement*, *manual*, *visual*, *ECG or EKG derived*, *impedance*, *thoracic pneumography*, *and hospital setting*. These terms were combined in strings using the Boolean operands “OR” and “AND” to specifically focus on studies that compared different methods to assess RR.

Studies were included if they met all of the following criteria: (a) included adult patients; (b) were a clinical trial or a comparative study that evaluated hospitalized patients; (c) compared VA of RR to IP and/or EDR; (d) were published between January 2000 and August 2020; and (e) were published in English.

**FIGURE 4:**
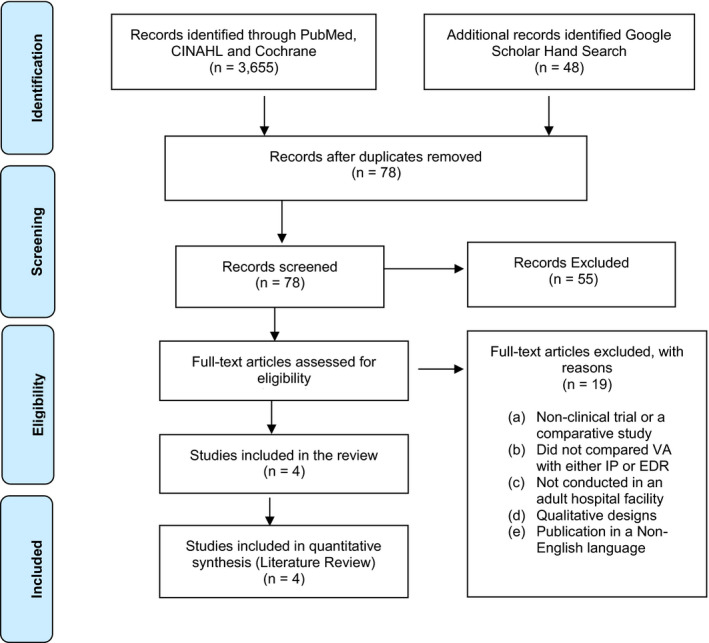
A diagrammatic representation of the literature search strategy using the PRISMA format (Moher et al., 2009)

The search strategy yielded 3607 studies identified in PubMed, 21 in CINAHL, 16 in Web of Science, and 11 in the Cochrane Library (Figure 4). An additional 48 studies were found in Google Scholar. After duplicates and articles not directly relevant to the topic were removed, the abstracts from 78 studies were evaluated. Of these 78 studies, full manuscripts for 23 studies were reviewed. After eliminating studies that did not meet our pre‐specified inclusion criteria, four studies are included in this systematic review. Of these four studies, 3 (75%) compared VA to IP (Chand et al., 2014; Granholm et al., 2016; Lovett et al., 2005) and 1 (25%) compared VA to EDR (Kellett et al., 2011).

The findings from this review are summarized in Table 1. Standardized criteria were developed to review the two groups of studies. Across both groups of studies, information was obtained on the author, year, purpose, study design, sample characteristics, study procedures and analysis methods, main findings, and strengths and limitations.

**TABLE 1:** Summary of the findings from studies that compared respiratory rates (RR) identified using visual assessment (VA), impedance pneumography (IP), and/or electrocardiographic‐derived (EDR) methods

Author, year, country, purpose, setting, and study design	Sample characteristics	Study procedures and methods of data analysis	Main findings	Strengths and limitations
VA compared to IP				
Author: Lovett et al. (2005) Country: USA Purpose: Measure the variability and accuracy of triage nurses' measurements of RR relative to criterion standard measurements and Evaluate the variability and accuracy of electronic measurements of RR recorded using a cardiac monitor equipped with transthoracic impedance (IP) Setting: urban teaching ED Design: Cross‐sectional	Sample size: 159 consecutive patients who presented to the ED Age (years) 18–29 = 34.0% 30–39 = 22.6% 40–49 = 14.5% 50–59 = 8.8% 60–69 = 7.5% 70–79 = 5.7% 80–89 = 1.3% NR = 5.7% Mean Age = 39.41 Female = 50.9% Hispanic = 41.5% White = 46.5%	Description of study procedures: Triage nurses' measurements of RR were recorded from the medical record Research Assistants (RAs) were trained in standardized methods to collect criterion standard measurements of RR. RAs observed respirations and auscultated RR at a single location for one minute. When auscultation could not be performed, observed RR was used in the analyses. RR using the IP method was captured at 60‐s intervals. Data analysis: Variability—was estimated by calculating the SD of each of the measures. Differences among the nurse, RA, and IP measures were evaluated using ANOVA. Sensitivity and specificity of triage nurses versus IP were cross‐tabulated measures against criterion standard measurements of respiratory values: Low = <12 breaths per minute Normal = 12–20 breaths per minute High = >20 breaths per minute Bland–Altman analyses were done that compared for—(a) triage nurses RR to criterion standard RR and (b) criterion standard RR to IP rates Agreement Bias 95% limits of agreement	Variability for triage nurses' measurements of RR (3.3) was significantly lower than for IP (4.1) and criterion standard (4.8, *p *< .01). Variability for IP measure was significantly lower than for criterion standard measure (*p *< .05) Accuracy of detecting bradypnea and tachypnea—neither triage nurses nor IP measures of RR were accurate in detecting bradypnea or tachypnea Bradypnea (<12 breaths/min) Nurse versus criterion measure ▪ Sensitivity = 0.00 (0.00–0.35) ▪ Specificity = 1.00 (0.97–1.00) IP versus criterion measure ▪ Sensitivity = 0.25 (0.07–0.59) ▪ Specificity = 0.98 (0.94–0.99) Tachypnea (>20 breaths/min) Nurse versus criterion measure ▪ Sensitivity = 0.38 (0.25–0.53) ▪ Specificity = 0.84 (0.75–0.90) IP versus criterion measure ▪ Sensitivity = 0.40 (0.28–0.55) ▪ Specificity = 0.86 (0.78–0.92) Agreement between triage nurses and criterion measure of RR was poor (95% limits of agreement −8.6 to 9.5) Agreement between IP and criterion measure of RR was poor (95% limits of agreement −9.9 to 7.5) Systematic bias was small for triage nurses' measurements of RR (+0.0) and electronic measurements of RR (−1.2)	Strengths ▪ Data collected in an ED during triage ▪ The criterion reference standard used for comparison ▪ Use of Bland–Altman analyses Limitations ▪ The majority of the patients were less than 39 years of age ▪ Triage nurses were aware that their assessments of RR were being collected ▪ Criterion measure of RR was obtained after the triage visit, not simultaneously with triage nurses' assessment of RR ▪ No inter‐rater reliability estimates were done with the RAs
Author: Chand et al. (2014) Country: India Purpose: Examine differences between VA and electronic (IP) measurements of vital signs in cardiac patients Setting: Advanced Cardiac Centre ICU Design: Comparative study	Sample size: 50 patients admitted in CTVS‐ICU and CCU CTVS‐ICU = 21 (42%) CCU = 29 (58%) Mean age (Years) =55.9 Females = 49.25 (range 25–58) Females = 16% Ethnicity = NR	Description of study procedures: VA—By floor RNs IP—By the cardiac monitor Four measurements of temperature, pulse, respiration, and blood pressure were recorded at 30‐min intervals, consecutively. The measurement of each vital sign was done simultaneously. Data analysis: Paired *t* test was used to evaluate for differences between the VA and IP methods The coefficient of variation was calculated to quantify the variation between the VA and IP measures	A total of 200 measurements were done using each method The mean difference in RR between the VA and IP methods was not significant (i.e., 0.015 (±1.16), *p* = .883) The coefficient of variation between the VA (26.25%) and IP (25.48%) was similar	Strengths ▪ Measurements made simultaneously Limitations ▪ Purposive sampling ▪ Type of physiologic monitor not reported ▪ Unclear if nurses were blinded to values obtained with the IP methods ▪ Small sample size ▪ Bland–Altman analyses were not performed
Author: Granholm et al. (2016) Country: Denmark Purpose: Evaluate the agreement between RR rates done using three methods (i.e., standardized approach, VA by ward staff, IP) Setting: Medical unit Design: Prospective, observational study	Sample size: 50 patients admitted to an acute medical unit Median age (years) =71.5 Female = 54% Ethnicity = NR	Description of study procedures: VA—Ward staff performed all assessments as usual. Data obtained from medical record IP—Sensium Vitals wireless patch measures RR, heart rate, and axillary temperature every 2 min Standardized approach—Trained researchers counted the patient's RR over 60 s. Patients were instructed to lie still and refrain from talking Data analysis: Bland–Altman analysis used to evaluate the agreement between the methods with 95% LOA and 95% CI	Agreement between standardized VA by researcher versus IP ▪ Mean difference was 0.3 b/m (95% CI −1.4 to 2.0 b/m) ▪ Lower and upper 95% LOAs were −11.5 b/m (95% CI −14.5 to −8.6 b/m) and 12.1 b/m (95% CI 9.2 to 15.1 b/m) respectively ▪ Large RR differences (>10 b/m) were found in three outliers (i.e., one obese patient with respiratory disease; one elderly patient with respiratory disease, atrial fibrillation, and prior cardiac surgery; one slim young patient with a non‐respiratory‐related infection) ▪ The mean difference after removing three outliers was −0.1 b/m (95% CI −0.7 to 0.5 b/m). Without outliers' differences were normally distributed Agreement between VA by ward staff versus IP ▪ Mean difference was 1.7 b/m (95% CI −0.5 to 3.9 b/m) ▪ Lower and upper 95% LOAs were −13.3 b/m 95% CI ▪ −17.2 to −9.5 b/m and 16.8 b/m (95% CI 13.0 to 20.6 b/m), respectively ▪ RR by ward staff was not normally distributed, with digit preferences of 16, 18, and 20 b/m	Strengths ▪ One trained researcher recorded the standardized approach ▪ The single paired measurement used for each patient minimized bias caused by within‐subject correlations Limitations ▪ No repeated measurements ▪ RR done by ward staff were obtained from the electronic health record, which could affect comparison with IP (i.e., inaccurate times recorded) ▪ Small sample size
VA compared to EDR				
Author: Kellett et al. (2011) Country: Ireland Purpose: Evaluate for the association between VA and EDR measured RR and their relationships to in‐hospital mortality Setting: Acute medical unit in a small rural hospital Design: Descriptive, correlational	Sample size: 377 acutely ill medical patients Mean age (years) – 68.3 ± 16.8 Alive = 67.9 (±17.0) Dead = 77.1 (±9.2) Sex = NR Ethnicity = NR	Description of study procedures: VA of RR was obtained by one of eight nurses on the patient's admission to the unit. Nurses were not given any instructions on how to measure or record RR. EDR: RR was obtained using a BT16/Piezoelectric belt for 5 min after admission. Data were transmitted to a separate computer system for subsequent analyses. Data analysis: Paired *t* tests were used to evaluate for differences in RR between VA and EDR Correlation coefficients were calculated for VA versus EDR measures of RR. Bland–Altman plots were done to evaluate the limits of agreement between the VA and IP measures of RR	The mean RR measured by VA (20.9 (±4.8) breaths/min) was significantly different from that obtained by EDR (19.9 (±4.5) breaths/min), *p *= .004 The correlation coefficient between VA and EDR was 0.50. Visual inspection of the scatter plots illustrated that RR obtained using VA clustered around rates of 18, 20, and 22 breaths/min. The RR rates obtained using EDR were more variable. Bland–Altman plots revealed that the 95% LOA between VA and EDR for RR were −8.2 and 10.3 breaths/min	Strengths ▪ Relatively large sample size Limitation ▪ Demographic and clinical characteristics of the sample (e.g., acuity level, use of medications) were not reported ▪ Only eight nurses participated in this study, and their characteristics were not reported ▪ Lack of standardization in the VA or RR ▪ Bland–Altman plots not included in the paper

Abbreviations: b/m, breaths per minute; CTVS‐ICU, cardiothoracic and vascular surgery‐intensive care unit; CCU, critical care unit; CI, confidence interval; CSM, criterion standard measurement; EDR, electrocardiographic‐derived respiration; IP, impedance pneumography; ED, emergency department; LOA, limits of agreement; NR, not reported; PACU, post anesthesia care unit; VA, visual assessment; RN, registered nurse; RR, respiratory rate; SD, standard deviation.

## 3 RESULTS

### 3.1 Results of the studies that compared VA to IP

#### 3.1.1 Description of the studies

All of the studies that compared the VA and IP methods were cross‐sectional descriptive studies (Chand et al., 2014; Granholm et al., 2016; Lovett et al., 2005). These studies were conducted in the United States, India, and Denmark. Sample sizes ranged from 50 (Chand; Granholm) to 159 (Lovett). Of the two studies that reported mean age (Chand and Lovett), the grand mean age was 45.6 years. Across the three studies, the grand mean percentage of females was 46.7%.

#### 3.1.2 Description of the study procedures

In all three studies, nurses' VA of RR was used for comparative purposes. In two of these studies (Granholm; Lovett), research staff were trained to provide an additional VA of RR that was used as the criterion standard measure. Visual assessment allows for observation of other breathing characteristics such as depth, skin color (i.e., cyanosis), or the use of accessory muscles that will indicate acute respiration distress. IP measures were captured using a cardiac monitor (Chand; Lovett) or a Sensium Vitals wireless patch (Granholm).

#### 3.1.3 Description of the methods used to assess the accuracy of VA to IP

Across these three studies (Chand et al., 2014; Granholm et al., 2016; Lovett et al., 2005), the analytical methods used to assess VA's accuracy compared with IP were extremely variable. In two studies (Chand et al., 2014; Lovett et al., 2005), paired analyses were done to evaluate variability between or among the measures. In one study (Lovett et al., 2005), sensitivity and specificity analyses were done for bradypnea and tachypnea. In two studies (Granholm et al., 2016; Lovett et al., 2005), Bland–Altman analyses were performed.

#### 3.1.4 Summary of major findings

The results of the comparative findings between the VA and IP methods were highly variable depending on the analytic method used. In Lovett et al., when comparative methods were used (e.g., analysis of variance), variability in the RR (not necessarily a good measure of accuracy) obtained by nurses using VA was lower than for either the criterion standard or IP measures. In Chand et al., no differences were found using paired *t* tests between the VA and IP methods. However, in both studies that used Bland–Altman analyses (Granholm; Lovett) the upper and lower levels of agreement (LOA) between the two methods were extremely poor.

### 3.2 Results of the study that compared VA to EDR

Only one study was found that compared the VA and EDR methods (Table 1) (Kellett et al., 2011). In this descriptive correlational study, VA of RR in 377 critically ill patients was done by one of eight unit nurses. EDR was compared with the RR derived from a BT16 Bluetooth acquisition system (Francesco Marazza, Milan, Italy) using a piezoelectric belt around the chest, which responded to changes in thoracic diameter. RR was obtained using this device within 5–10 min after admission. Using paired t tests, significant differences in RR were found between the two methods. In addition, using Bland–Altman analyses, the LOAs between the two methods were poor. Of note, visual inspection of the scatter plots determined that RR obtained using VA centered around rates of 18, 20, and 22 breaths per minute. In contrast, the RR obtained using EDR were more variable.

## 4 DISCUSSION

While designed to be a systematic review that compared the accuracy, strengths, and limitations of VA, IP, and EDR methods to measure RR, only four studies were identified (Chand et al., 2014; Granholm et al., 2016; Kellett et al., 2011; Lovett et al., 2005). Of note, none of these studies compared all three methods in the same sample of patients. The remainder of this discussion will provide a synthesis of the findings, discuss the strengths and limitations of the three methods, and suggest directions for future research.

One of the limitations of the current studies was the choice of the “gold standard” or reference group that was used for comparative purposes. While all four studies used VA by nurses to determine RR (Chand et al., 2014; Granholm et al., 2016; Kellett et al., 2011; Lovett et al., 2005), it is well known that these results are not standardized and, as noted in one study (Granholm et al., 2016), were not normally distributed and were prone to having even numbers reported (e.g., 18, 20). In the two IP studies that used trained researchers to perform VA of RR for comparative purposes, (Granholm et al., 2016; Lovett et al., 2005), the findings are inconclusive. A major limitation of these two studies is that the training procedures for the research staff to ensure inter‐rater reliability were not described. Finally, it is important to note that nurses often count breaths using a 30 s time window and then multiple this value by two to obtain the number of breaths/minute. This short time period could explain the low variability of nurse RR when compared to device‐driven methods that measure RR continuously over longer time intervals.

An equally important consideration in the evaluation of the comparability of methods is the choice of statistical tests. Three of the four studies used the Bland–Altman analysis to evaluate for agreement between VA of RR and the IP (Granholm et al., 2016; Lovett et al., 2005) and EDR (Kellett et al., 2011) methods. Compared with the calculation of a correlation coefficient, the Bland–Altman analysis describes the agreement between two quantitative measures by constructing LOA. These statistical limits are calculated using the mean and the standard deviations of the differences between the two measurements (Giavarina, 2015) However, it should be noted that only a clinician, who will use the test results, can determine whether the LOA are or are not acceptable (Doğan, 2018). In all three studies (Chand et al., 2014; Granholm et al., 2016; Lovett et al., 2005), the upper and lower LOA between VA and the IP and EDR methods were very poor.

Several study limitations contribute to these significant discrepancies including relatively small sample sizes, lack of inter‐rater reliability assessments, cross‐sectional designs, and heterogeneity in patient samples. Given the clinical need to have accurate counts of RR in critical care settings (Brekke et al., 2019; Kelly, 2018; Mochizuki et al., 2017), additional research is warranted on the use of both the IP and EDR methods. Future studies need to develop rigorous research protocols that included: training and evaluation of the inter‐rater reliability of the research staff who perform the VA of RR; power calculations to determine appropriate sample sizes; pre‐specified criteria for acceptable LOA; conducting experiments to determine acceptable and clinically meaningful LOA for various clinical conditions (e.g., tachypnea, bradypnea, normal RR); and methods for dealing with changing conditions during recording such as changes in body position (i.e., side lying, flat, upright), which were not addressed in the studies examined.

As noted in the Introduction, accurate, real‐time assessments of RR, which use physiologic data and are integrated into the critical care environment, may contribute to earlier detection of clinical deterioration (Brekke et al., 2019; Kelly, 2018; Mochizuki et al., 2017; Subbe & Kinsella, 2018). Given the paucity of evidence, the remainder of this discussion will describe the advantages and disadvantages of the VA, IP, and EDR methods to improve the earlier detection of deleterious changes in RR (see Table 2). While VA is easy to perform, does not require any additional equipment, involves human interaction, and allows a clinician to evaluate a number of breathing characteristics (e.g., depth, skin color), it is not the ideal method for critically ill patients. For example, VA is time‐consuming and prone to numerous omissions (Ansell et al., 2014; Hogan, 2006). In addition, inaccurate measurements can occur because of environmental distractions and patient movement (Goldhill et al., 2005; Kamio et al., 2018; Yanovski et al., 1996). However, the major limitation in the critical care setting is that because VA of RR is done at prescribed intervals (e.g., every 30 min), dynamic changes in RR are missed.

**TABLE 2:** Comparison of the strengths and limitations of the visual assessment (VA), impedance pneumography (IP), and ECG‐derived respiration (EDR) methods for assessment of respiratory rate

Methods	Strengths	Limitations
VISUAL	Traditional method to assess RR Easy and safe to perform Breathing characteristics (e.g., depth, accessory muscles, skin color) can be assessed	Time‐consuming for clinicians Numerous omissions and guessed measurements (Cooper et al., 2014) Low precision and/or variability because respiratory rate is often counted for 30 s, and then, the value is multiplied by two to get the number of breaths/minute VA is a snapshot of a patient's RR at prescribed intervals (e.g., every 30 min). Acute changes and early identification of patient deterioration can be missed
IP	Simpler, less time‐consuming than VA Safe to use Continuous measurement of RR Coherence analysis concluded that IP is more reliable than EDR (Ernst et al., 1999; Houtveen et al., 2006)	Studies found that the IP method was prone to erratic artifacts, false‐positive readings and was sensitive to motion and cardiac artifacts (Drew et al., 2014; Khambete et al., 2000; Młyńczak & Cybulski, 2012; Młyńczak et al., 2015; Seppa et al., 2010; Vuorela et al., 2010) A device's internal impedance, such as cables and wires, can be a source of measurement error (Landon, 2002) IP can generate false positives from movement and interruptions by the examinee and affect the readings and values (Krapohl & Shaw, 2015) IP method is influenced by behaviors that occur naturally (e.g., talking, coughing) (Krapohl & Shaw, 2015) IP is predisposed to signal degeneration with body position changes because the thoracic signal depends on posture, making it difficult to evaluate tidal volume (Landon, 2002)
EDR	The EDR algorithm can be added to existing ECG to extract respiratory signals from the ECG signal without new transducers, devices, or accessories required for monitoring (Charlton et al., 2016) Continuous monitoring and non‐invasive (Charlton et al., 2016) The sensitivity and specificity of the EDR algorithm to identify RR were high (99%/97%) in cardiac patients compared with other methods (Babaeizadeh et al., 2012) Alterations in the RR are easily detected	RSA aspect weakens with aging, which may lead to inaccurate measurements in older individuals. Patient movement and noise can cause artifacts and lead to inaccurate values. Method lacks validation in the hospital setting. EDR measurement can be affected by the natural decline in RSA, as well as arrhythmias (e.g., atrial fibrillation) and the effects of medications that affect heart rate and rhythm

**FIGURE 5:**
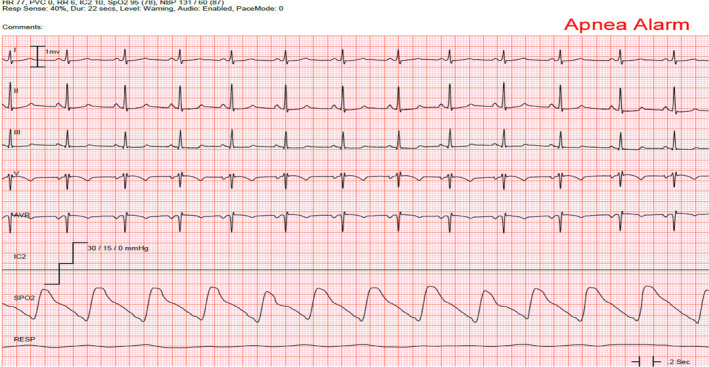
False apnea alarm in an intensive care unit patient measured using the impedance method. The respiratory waveform (bottom waveform labeled “RESP”) is essentially a flat line. Therefore, respiratory rated calculated using the impedance method alarmed for apnea. The monitor default setting for apnea is cessation of breathing for >20 s. However, this patient was not in acute respiratory distress at the time of this alarm. Note at the top of the alarm tracing is an erroneous respiratory rate (RR) of 6 breaths/min, yet the oxygen saturation measure from the Sp02 probe is 95%

The major advantages of the IP include that it is safe and simple to use; it is available in cardiac monitors; and it provides a continuous measurement of RR. However, signal interruptions and patient movement can affect the characteristics of the respiratory waveform and subsequent calculation of RR (Drew et al., 2014; Gupta, 2011). An example of this limitation is found in a study that reported 161,931 unique RR type alarms (i.e., RR parameter high/low, or apnea) from adult patients in the intensive care unit that used IP in their bedside monitors (Drew et al., 2014). As shown in Figure 5, a large proportion of the alarms were found to have flat RR waveforms in patients who were known to be breathing adequately, were not in respiratory arrest, and/or were on a ventilator.

The number of false alarms generated using the IP method is problematic because it interrupts nursing workflow unnecessarily and compounds the alarm fatigue problem. Another limitation of the IP method is that the various components of the impedance device (e.g., wires, skin electrodes and cables) can be sources of measurement error (Landon, 2002). Of note, while the IP method is widely accepted, in one review (Landon, 2002) it was noted that non‐respiratory motion and cardiac artifact can influence the accuracy of the readings (Landon, 2002). Cardiac artifact occurs when a pulsatile volume of blood moves through the aorta during each heartbeat; thus, changing the thoracic impedance measured by IP. This may appear as low amplitude oscillations at the heart rate of the patients and be superimposed on the IP signal and could be inadvertently interpreted as breaths, leading to overestimation of RR. Newer IP algorithms have been developed to minimize the influence of cardiac artifact (Lu et al., 2019).

While not as well studied in the clinical setting, the EDR method has numerous advantages (Kellett et al., 2011). Like the IP method, it is non‐invasive, it provides continuous assessment of RR, which means acute alterations in RR are easily detected. In addition, the EDR algorithm could be added to existing bedside monitors to extract respiratory waveforms from the ECG signal (Charlton et al., 2016). With this method, the detection and measurement of QRS complexes are comparatively impervious to noise and muscle artifact, making it an ideal waveform to use to derive RR (Helfenbein et al., 2014; Mazzanti et al., 2003). In addition, compared with IP, direct measurements of QRS amplitude are more highly correlated with tidal volume and the amplitude displacement caused by the rise and fall of chest movement, which may be more suitable for the identification of RR (Helfenbein et al., 2014).

In terms of limitations, similar to the IP method, device failure can occur. In addition, EDR measurement can be affected by the natural age‐related decline in RSA, as well as arrhythmias (e.g., atrial fibrillation) and the effects of medications that affect heart rate and rhythm (Helfenbein et al., 2014). Finally, patient movement can cause artifacts and lead to inaccurate assessments of RR. While this method holds promise, additional research is warranted that compares the accuracy of VA, IP and EDR in the same sample of critically ill patients.

### 4.1 Limitations of this review

The primary limitation of this review is the paucity of research on this topic. Given that only three studies compared the VA and IP methods (Chand et al., 2014; Granholm et al., 2016; Lovett et al., 2005) and only one compared VA to EDR (Kellett et al., 2011), no definitive conclusions could be drawn about the accuracy of these continuous device‐driven methods. In addition, given the paucity of the research and heterogeneity of the small number of studies included, a meta‐analysis could not be performed.

## 5 CONCLUSIONS

Given the importance of accurate and frequent RR assessment in the fast‐paced critical care environment, methods that take advantage of available physiologic data are warranted. Given the promise, but limitations of both the IP and EDR methods, future research needs to focus on making refinements to these algorithms and/or developing new algorithms that are easily integrated into existing physiologic devices used in the critical care environment. The use of a combined approach that utilizes the strengths of both IP and EDR may provide more precise and accurate results (Helfenbein et al., 2014). However, the optimal approach to combining these methods warrants additional investigation. Future studies need to include diverse patient populations with a variety of clinical conditions and employ the most robust analytic methods. This line of scientific inquiry will result in a clinically useful method to detect dynamic and acute changes to RR in critically ill patients who may require interventions to avert untoward outcomes.

## CONFLICTS OF INTEREST

The authors have no conflicts of interest to declare.

## AUTHOR CONTRIBUTIONS

LKB contributed to literature search; LKB, CM, MP, XH, and GR contributed to study design; LKB, CM, and MP contributed to data analysis; LKB, CM, and MP contributed to manuscript preparation; LKB, CM, MP, XH, and GR contributed to review of manuscript.

## ETHICAL APPROVAL

This is a non‐data based paper, rather a systematic review; hence, there are no data to share.

## Data Availability

No data available.
